# Revealing the Potency of Growth Factors in Bovine Colostrum

**DOI:** 10.3390/nu16142359

**Published:** 2024-07-21

**Authors:** Yalçın Mert Yalçıntaş, Hatice Duman, Jose M. Miranda López, Alicia C. Mondragón Portocarrero, Mauro Lombardo, Farid Khallouki, Wojciech Koch, Matteo Bordiga, Hesham El-Seedi, António Raposo, Jose Luiz de Brito Alves, Sercan Karav

**Affiliations:** 1 Department of Molecular Biology and Genetics, Çanakkale Onsekiz Mart University, Canakkale 17000, Türkiye; yalcinmertyalcintas@stu.comu.edu.tr (Y.M.Y.); hatice.duman@comu.edu.tr (H.D.); 2 Laboratorio de Higiene Inspección y Control de Alimentos, Departamento de Química Analítica, Nutrición y Bromatología, Universidade de Santiago de Compostela, Campus Terra, 27002 Lugo, Spain; josemanuel.miranda@usc.es (J.M.M.L.); aliciamondragon@yahoo.com (A.C.M.P.); 3 Department for the Promotion of Human Science and Quality of Life, San Raffaele Open University, Via di Val Cannuta, 247, 00166 Rome, Italy; mauro.lombardo@uniroma5.it; 4 Team of Ethnopharmacology and Pharmacognosy, Biology Department, Faculty of Sciences and Techniques, Moulay Ismail University of Meknes, Errachidia 50050, Morocco; f.khallouki@fste.umi.ac.ma; 5 Chair and Department of Food and Nutrition, Faculty of Pharmacy, Medical University of Lublin, 4a Chodźki Str., 20-093 Lublin, Poland; kochw@interia.pl; 6 Department of Pharmaceutical Sciences, Università del Piemonte Orientale, Largo Donegani 2, 28100 Novara, Italy; matteo.bordiga@uniupo.it; 7 Chemistry Department, Faculty of Science, Islamic University of Madinah, P.O. Box 170, Madinah 42351, Saudi Arabia; elseedi_99@yahoo.com; 8 CBIOS (Research Center for Biosciences and Health Technologies), Universidade Lusófona de Humanidades e Tecnologias, Campo Grande 376, 1749-024 Lisboa, Portugal; antonio.raposo@ulusofona.pt; 9 Department of Nutrition, Health Science Center, Federal University of Paraíba, João Pessoa 58051-900, PB, Brazil; jose.luiz@academico.ufpb.br

**Keywords:** colostrum, growth factors, immune responses, health benefits, nutritional supplements

## Abstract

Colostrum is a nutritious milk synthesized by mammals during the postpartum period, and its rich bioactive components has led to a global increase in the consumption of bovine colostrum as a supplement. Bovine colostrum contains key components such as immunoglobulins, oligosaccharides, lactoferrin and lysozyme. It is a special supplement source due to its natural, high bioavailability and high concentrations of growth factors. Growth factors are critical to many physiological functions, and considering its presence in the colostrum, further research must be conducted on its safe application in many bodily disorders. Growth factors contribute to wound healing, muscle and bone development, and supporting growth in children. Additionally, the molecular mechanisms have been explored, highlighting the growth factors roles in cell proliferation, tissue regeneration, and the regulation of immune responses. These findings are crucial for understanding the potential health effects of bovine colostrum, ensuring its safe use, and forming a basis for future clinical applications. This review article examines the growth factors concentration in bovine colostrum, their benefits, clinical studies, and molecular mechanisms.

## 1. Introduction

Colostrum is a pre-milk substance that is essential for the health and development of newborn mammals. This special pre-milk offers various positive effects thanks to its bioactive components. For example, it enhances athletic performance, aids in the healing of muscle injuries, helps with the development of immunity in newborns, and promotes muscle mass gain [1]. Colostrum’s composition closely resembles that of blood and differs markedly from milk. It contains a range of nutrients, including biologically active compounds and proteins, lipids, lactose, and vital fatty acids [2].

The various bioactive components of colostrum positively affect the body in different ways. For example, growth factors play a role in important metabolic activities such as wound healing, bone and muscle development, and cell proliferation [3,4,5].

**Figure 1:**
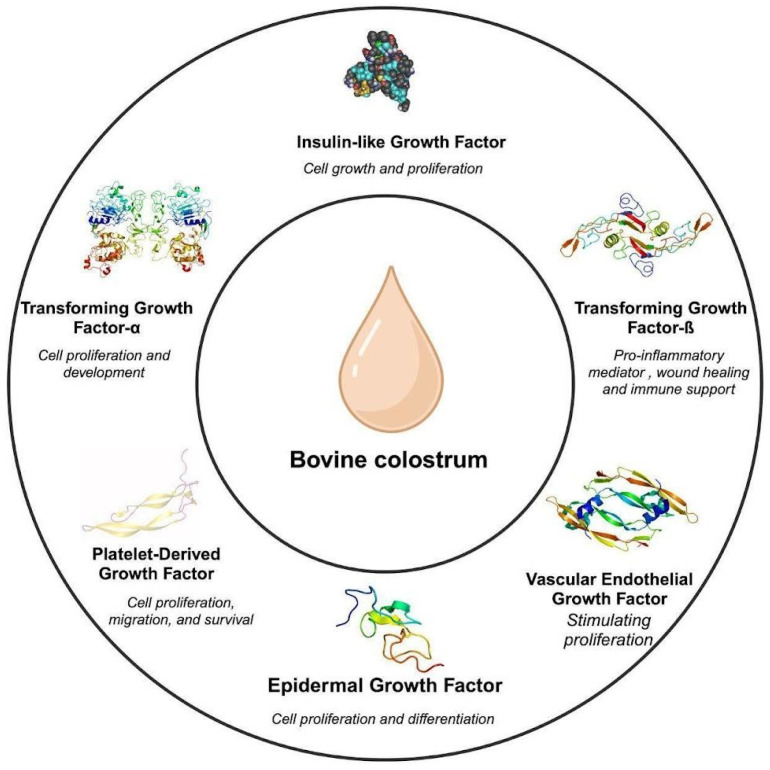
Growth factors in bovine colostrum [2,9].

Growth factors can vary in concentration depending on their source. Both regular milk and colostrum contain many peptide growth factors that help mammalian cells grow and differentiate. A study reported that within the first 10 h after birth, colostrum contains the optimal concentration of growth factors, shown in Table 1, although this timeframe may vary depending on the source and environmental factors [6]. The two main growth factors, insulin-like growth factors 1 and 2 (IGF-1 and 2) and transforming growth factors alpha and beta (TGF-A and B), are only found in colostrum (Figure 1). These growth factors exhibit remarkable biochemical attributes that contribute to muscle repair and wound healing [7]. Consuming bovine colostrum (BC) may be more effective compared to taking growth factor supplements orally, because BC contains a variety of bioactive components that work synergistically to promote health Also, applying growth factor supplementation topically and systemically rather than orally increases its effectiveness; a study reported that growth factors are degraded by digestive enzymes [8]. In this review article, various types of growth factors found in BC and their health benefits were examined.

**Table 1:** Growth factor concentrations and types in BC.

Growth Factor	Concentration in BC (ng/mL)	Concentration in Bovine Milk(ng/mL)	Properties	Reference
EGF	324.2	155 (pasteurized milk)	Cell proliferation, survival, and differentiation	[10,11]
IGF-1	870	150	Cell growth and proliferation	[6,12]
IGF-2	206	2–6	Regulates cell proliferation and survival	[12,13,14]
TGF-β	100.7	4.3 (pasteurized milk)	Pro-inflammatory mediator, stimulating the activation and migration of immune cells and wound healing	[15,16]
TGF-α	200	-	Cell proliferation, differentiation, and development	[17,18]
PDGF	-	-	Cell migration, proliferation, and survival	[19]

## 2. Exploring the Growth Factors: Types and Molecular Mechanisms

Bovine colostrum is a rich supplement in terms of growth factor concentrations and the types it contains. Clinical studies have demonstrated the effects of administering growth factors derived from bovine milk or bovine colostrum in various conditions such as diseases and injuries [1,9].

Growth factors have an important role in fundamental cellular processes, including growth, proliferation, differentiation, and survival. They achieve this intricate control by binding to specific receptors on the cell surface, initiating a cascade of intracellular signaling events. This intricate signaling network stimulates the regulation of gene expression and cellular functions, directing the cell’s behavior. Epidermal Growth Factor (EGF), TGF-β, platelet-derived growth factor, and IGFs are the main types of growth factors.

### 2.1. Epidermal Growth Factor (EGF)

EGF is a compact protein consisting of approximately 53 amino acids categorized within the EGF ligand family [20]. Members of this ligand family, including EGF, exhibit structural resemblances and interact with a group of receptors known as ErbB receptors, among which EGFR stands out as extensively studied [21]. Numerous cell types, including fibroblasts, endothelial cells, and epithelial cells, produce EGF [22]. Typically, it is generated as a transmembrane precursor and subsequently undergoes cleavage by specific proteases. This process liberates the mature, active EGF ligand into the extracellular environment [23]. EGF exhibits high-affinity binding to the extracellular domain of EGFR. This binding pocket within EGFR is formed by loops and β-sheets, creating a specific docking site for EGF [24]. When EGF binds to EGFR, the receptor’s conformation changes significantly, exposing previously hidden areas and facilitating the initiation of further signaling pathways [24]. This binding triggers the receptor to form dimers and phosphorylate tyrosine residues in its cytoplasmic domain [10]. This phosphorylation activates pathways including Ras/MAPK and PI3K/Akt, which control cell proliferation, survival, and differentiation [25,26]. It is worth noting that EGF is just one member of a larger EGF ligand family. Each ligand may have slightly different binding affinities and can activate distinct signaling pathways within the cell depending on the specific ErbB receptor it interacts with [27]. This adds complexity and potential for tailored cellular responses. The existence of EGF-like domains in other proteins suggests possible communication between various signaling pathways that involve the ErbB receptor family [28].

### 2.2. Transforming Growth Factor Beta (TGF-β)

The TGF-β superfamily, of which there are three members (TGF-β1, TGF-β2, and TGF-β3), includes the cytokine TGF-β. Within cells, TGF-β molecules are first generated as precursor proteins [29]. These precursors undergo cleavage to form mature TGF-β ligands, which remain bound to a latency-associated peptide (LAP) in a non-covalent manner [30,31]. This association prevents the premature activation of TGF-β signaling [31]. The release and activation of the TGF-β-LAP complex require additional processing, which can happen through different mechanisms such as proteolytic cleavage or interaction with integrins (cell surface receptors) or thrombospondin-1 (a matrix protein) [32]. After activation, the mature TGF-β ligand exhibits strong binding to specific transmembrane TGF-β receptors (TGFBRs) located on the cell surface [33]. The TGFBRs undergo a conformational shift brought on by ligand interaction, which causes them to dimerize. Signal transduction requires this dimerization, and the dimerized TGFBRs phosphorylate particular Smad proteins (Smad2 and Smad3) inside the cell [33]. Subsequently, these phosphorylated Smads form heterodimers with Smad4, a shared partner [33]. Once within the nucleus, the Smad complexes interact with co-activators or DNA to regulate gene expression and trigger biological reactions [34].

### 2.3. Platelet-Derived Growth Factor (PDGF)

**Figure 2:**
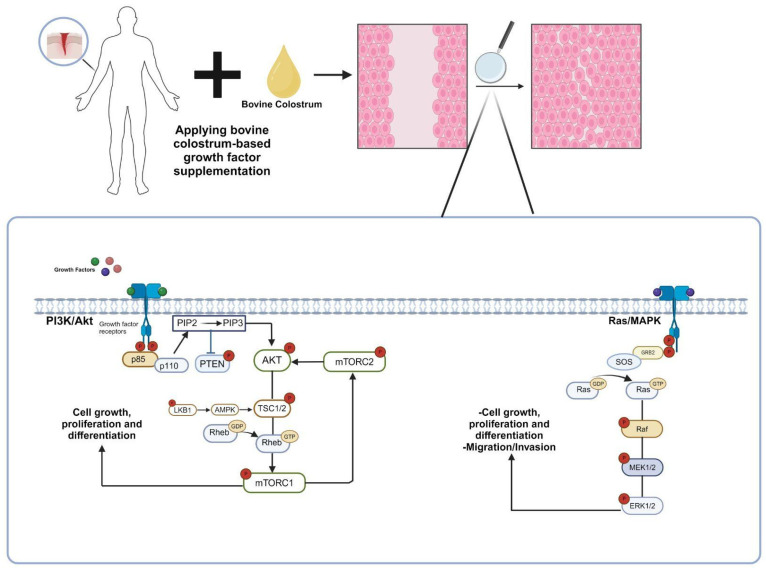
The molecular mechanism of the contribution of growth factors found in bovine colostrum to wound healing. Growth factors found in bovine colostrum stimulate the Ras/MAPK and PI3K/Akt signaling pathways through receptor binding, promoting the proliferation and differentiation of cells in the affected area. Consequently, the wound-healing process is accelerated [25,26]. (p85: regulatory subunit of phosphatidylinositol 3-kinase, p110: catalytic subunit of phosphatidylinositol 3-kinase, PTEN: phosphatase and tensin homolog, Akt: protein kinase B, PIP2: phosphatidylinositol (4,5)-bisphosphate, PIP3: phosphatidylinositol (3,4,5)-trisphosphate, TSC: tuberous sclerosis complex, Rheb: Ras homolog enriched in brain, mTORC1: mechanistic target of rapamycin complex, LKB1: liver kinase B1, AMPK1: AMP-activated protein kinase, GRB2: growth factor receptor-bound protein 2, SOS: Son of Sevenless, Ras: rat sarcoma, Raf: rapidly accelerated fibrosarcoma, MEK: mitogen-activated protein kinase, ERK: extracellular signal-regulated kinase).

PDGF is present in four isoforms, namely, PDGF-AA, PDGF-AB, PDGF-BB, and PDGF-DD, each composed of homodimers or heterodimers of A and B chains [19]. These isoforms attach to particular PDGF receptors (PDGFRs) on the cell surface. PDGFRα and PDGFRβ are two types of PDGFRs that are members of the receptor tyrosine kinase (RTK) family [19]. PDGF-AA predominantly interacts with PDGFRα, whereas PDGF-BB binds to both PDGFRα and PDGFRβ. PDGF-AB can bind to either receptor. Upon the binding of the ligand, the PDGFR undergoes a conformational change [19]. Ligand binding triggers the activation and dimerization of PDGFRs, involving homodimers (α-α or β-β) or heterodimers (α-β) [19]. This dimerization process provides a binding site for intracellular signaling molecules, initiating downstream signaling pathways. PDGFR activation triggers multiple downstream signaling pathways, such as the Ras-MAPK (Figure 2), PI3K-Akt (Figure 2), and PLCγ pathways, depending on the specific receptor and cellular context [35]. Eventually, these pathways result in a variety of biological reactions, such as cell migration, proliferation, and survival.

### 2.4. Insulin-Like Growth Factors (IGFs)

IGFs bind to specific cell surface receptors, primarily the IGF-1 receptor (IGF1R), and to a lesser extent, the insulin receptor (IR) [36]. IGF1R is a transmembrane receptor tyrosine kinase (RTK) and associated with several intracellular pathways, including the RAS-MAPK and PI3K-AKT (Figure 2) [37]. Certain tyrosine residues in the receptor’s cytoplasmic domain become autophosphorylated when IGF binds, causing the receptor to undergo a conformational shift [38]. The phosphorylated tyrosine residues on IGF1R serve as docking sites for adaptor proteins containing Src homology 2 (SH2) domains [39]. Upon binding to these sites, adaptor proteins, including Grb2 and Shc, become activated, starting downstream signaling pathways [40]. This leads to the activation of major signaling pathways, including the mitogen-activated protein kinase (MAPK) pathway, which regulates cell proliferation and survival, and the PI3K/Akt/mTOR pathway (Figure 2), which is involved in cell growth and metabolism [12].

It should be noted that the molecular mechanisms of growth factors, as indicated in Figure 2 and discussed in the text, can lead to side effects if used improperly. For instance, uncontrolled cell proliferation may contribute to tumor formation. Therefore, consulting experts in the field of growth factor supplementation is crucial.

## 3. Clinical Trials and Effects of Growth Factors on Various Diseases

Growth factors are essential for many physiological functions, including accelerating the healing of wounds by stimulating tissue repair and cell proliferation. Additionally, the reason for using BC as a source of growth factors is its high bioavailability and synergistic activity. Research studies have reported results supporting this phenomenon [41,42]. Their ability to promote the regeneration of injured tissues makes them promise for the treatment of musculoskeletal injuries and disorders like osteoarthritis. Furthermore, growth factors strengthen the body’s defenses against infections and illnesses by increasing the generation and function of immune cells. Studies indicate that their advantages surpass immune support, pointing to wider uses in maintaining health and managing illnesses. In this part, molecular mechanisms of various types of GF and their applications are discussed.

### 3.1. Wound Healing

EGF and TGF-β1 are critical factors in wound healing, each playing distinct but complementary roles. EGF stimulates granulation tissue development, decreases inflammation, and encourages re-epithelialization to speed up wound closure [43]. In a clinical study, it was reported that EGF derived from bovine colostrum plays a role in the healing process of intestinal epithelial wounds [44]. On the other hand, TGF-β1 initially acts as a pro-inflammatory mediator, stimulating immune cell activation and migration. As the healing process progresses, TGF-β1 transitions to an anti-inflammatory role crucial for tissue repair and inflammation resolution [15].

Adding to these critical growth factors, PDGF orchestrates essential processes in wound healing. PDGF functions as a powerful chemoattractant, drawing fibroblasts, neutrophils, monocytes, and smooth muscle cells to the injury site [3,45]. This recruitment is vital for initiating the repair process and laying the foundation for tissue regeneration. Additionally, PDGF activates macrophages, triggering them to release growth factors that further stimulate healing mechanisms [3,45]. Furthermore, PDGF promotes the proliferation of fibroblasts, increasing their numbers within the wound area. Fibroblasts are crucial for producing the extracellular matrix (ECM), which provides structural support and scaffolding for tissue repair [46]. By enhancing ECM production, PDGF significantly contributes to the formation of new tissue and the restoration of tissue integrity.

The IGF family, in addition to EGF, TGF-β, and PDGF, plays a major role in the intricate series of processes involved in wound healing. IGFs stimulate epithelial cell migration and proliferation, which improves re-epithelialization, an essential stage of the healing process that replaces the skins or mucosal protective layer over the wound site [47,48]. Furthermore, IGFs stimulate the proliferation of fibroblasts, which are pivotal in producing collagen and other components of the ECM [48]. This activity is crucial for providing structural support to the healing tissue and promoting wound contraction. Understanding the collaborative roles of EGF, TGF-β1, IGF, and PDGF underscores their potential as targeted therapies to optimize wound healing outcomes. Using a mouse excisional wound model, it was shown that treatment with bovine milk extracellular vesicles (EVs) promoted re-epithelialization, activated angiogenesis, and enhanced extracellular matrix maturation [49]. Together, they effectively manage inflammatory responses, promote tissue regeneration, and accelerate the overall healing process.

### 3.2. Gastrointestinal Health

IGF-1 plays a critical role in enhancing intestinal cell growth and supporting overall gut health. Research has demonstrated its ability to increase nutrient absorption, promote mucosal growth, and boost intestinal weight in studies involving piglets [50,51]. These findings suggest that IGF-1 could be a beneficial therapeutic option for improving intestinal structure and function. IGF-1 also shows promise in protecting against cytokine-induced apoptosis, which helps to maintain the integrity of intestinal epithelial cells and reduces damage during inflammation [52]. This dual action of promoting growth and protecting against inflammation underscores IGF-1’s potential in enhancing overall gut health. In summary, IGF-1 emerges as a valuable candidate for future research and potential therapies aimed at enhancing intestinal health through its role in cell growth stimulation and protective effects against inflammation. Several reports have evaluated the delivery of TGF-β in foods, in enteral formulas, or directly by gavage in animal models of mucosal inflammation [53]. The preclinical and clinical findings show that TGF-β supplementation decreases mucosal and systemic inflammation in Crohn’s disease and ulcerative colitis [53].

### 3.3. Cancer

In oncology, while EGF is essential for normal cellular functions, its overexpression or dysregulation is associated with various cancers. As a result, EGF and its receptor (EGFR) are targets for cancer therapies, with several EGFR inhibitors having been developed to block the proliferative signals in cancer cells [54]. In the context of cancer, IGF-2 is often overexpressed in various tumors, contributing to tumor growth and progression [55]. It plays a crucial role in oncogenesis due to its capacity to stimulate cell division and prevent apoptosis. Thus, targeting the IGF-2 signaling pathway is being investigated as a possible therapeutic approach for the treatment of cancer [56,57]. However, the adaptability of TGF-β2 goes far beyond bone production, involving a variety of biological processes such as immunological regulation, wound healing, cell development, inflammation management, and even cancer metastasis [58,59,60].

### 3.4. Bone and Muscle Health

IGF-1 is a crucial peptide hormone involved in the growth and developmental processes of mammals [61]. This growth factor system influences the vasculature through a variety of physiological effects, operating via both endocrine and autocrine/paracrine mechanisms [62]. IGF-1 is a hormone that supports and stimulates growth, and studies have shown that it increases osteoblastic activity [63]. Additionally, from a different perspective, exercise or training activates the GH and IGF-1 axis. Conversely, exercise also increases catabolic pro-inflammatory cytokines, such as interleukin-6 (IL-6) [64]. The anabolic GH and IGF-1 axis are crucial because its activation after exercise enhances muscle growth and repair, increases protein synthesis, and promotes fat metabolism, leading to improved muscle strength, reduced body fat, and overall better physical performance [64].

Also, one study aimed to improve muscle injury recovery in elderly people [65] (Table 2). Muscle stem cells become less common as people age, but the ones that remain can still regenerate in a manner similar to those of youth [65]. The research showed that changes in the muscle environment with age reduce the regenerative capacity of these cells. IGF-2 levels were observed to be lower in the regenerated muscles of older mice based on protein analysis. By promoting stem cell proliferation and blood vessel creation while decreasing the formation of fat cells, pro-IGF-2 supplementation of elderly mice resulted in enhanced muscle regeneration [65].

### 3.5. Neurological and Mental Health

Growth factors play a crucial role in regulating cellular activities during both the embryonic and postnatal stages. Minor alterations in the expression patterns of these factors can significantly impact brain development. These initial changes can lead to various neuroanatomical and biochemical variations observed in the later stages of brain maturation [89]. Abnormal levels of growth factors have been implicated in the development and clinical presentation of multiple psychiatric disorders [89].

IGF-2 is a critical regulator of cellular processes such as proliferation, migration, differentiation, and apoptosis (programmed cell death) [90]. IGF-2 exerts its cellular effects through three distinct receptors: IGF-1 receptor (IGF-1R), insulin receptor (IR), and IGF-2 receptor (IGF-2R) [91]. Binding to these receptors triggers the activation of critical intracellular signaling pathways, including PI3K/Akt and MAPK, and these pathways serve as molecular keys regulating cellular growth, survival, and metabolism (Table 2) (Figure 2) [91]. While IGF-2 was traditionally seen as a fetal growth driver, new research has revealed its continued significance in adulthood [92]. Scientists have found high levels of IGF-2 gene expression in the central nervous system, where it appears to play a critical role in memory consolidation, especially involving the hippocampus [93]. Intriguingly, IGF-2 may be linked to memory-related disorders like schizophrenia, depression, etc. While its exact role remains unclear, further research holds promise for new treatments and diagnoses in these critical conditions [92].

### 3.6. Eye Health

The fluid inside the human eye, called aqueous humor, helps nourish the parts like the cornea and lens. This fluid contains special signaling molecules, and TGF-β2 is the most common one [94]. The concentration of a signaling molecule known as TGF-β2 increases in the fluid of the eye during certain eye conditions. This occurs in conditions such as proliferative vitreoretinopathy (PVR), diabetic retinopathy, and glaucoma [95]. Studies in mice lacking specific TGF-β isoforms have revealed their distinct roles in eye development [95,96]. While deficiencies in TGF-β1 or TGF-β3 do not cause eye problems, mice lacking TGF-β2 exhibit several malformations. These include a thinner cornea missing its inner cell layer, an undeveloped front chamber, an immature retina, and blood vessels persisting in the normally clear vitreous gel. All isoforms of TGF-β, including TGF-β3, are demonstrably expressed during fetal eye development. The specific pattern of TGF-β3 expression observed in the ganglion cell layer, photoreceptor layer, and choriocapillaris during the second trimester suggests its potential involvement in several critical processes. These processes likely include morphogenesis, development, and/or differentiation of the fovea, a specialized region responsible for central vision [97].

## 4. Growth Factors as Health Products

Given their therapeutic promise in wound healing, tissue regeneration, and disease management, growth factors—naturally occurring proteins—have attracted significant attention in the production of health products. These proteins can stimulate cellular growth, proliferation, and differentiation.

Growth factors can be presented in various forms in the medical and dietary supplementation fields. As shown in Table 3, different types of growth factors can be incorporated as the main active ingredients in these products. These products can be used as supportive treatments for conditions such as wound formation due to chronic diseases, tissue damage, growth disorders, hormonal imbalances, and skin aging. Healthcare professionals may use growth factors not only as supportive treatments, but also as therapeutic drugs. The wide variety of growth factors and their unique properties allow for the discovery of new applications beyond the current usage areas. In particular, the ability of growth factors to promote tissue regeneration holds the potential for broader therapeutic applications through advanced studies. In addition to medical treatments, these products can also be used to enhance performance and overall well-being in healthy individuals [98].

Growth factor therapy has several limitations. A key issue is the short half-life of growth factors in tissues, requiring a continuous angiogenic stimulus to maintain new vessel growth [105]. Systemic expression can lead to harmful side effects in distant organs, such as promoting tumor growth or arthritis [105]. For instance, VEGFs can increase vascular permeability and edema, with pericardial effusion being a dose-limiting side effect [106]. Therefore, consulting medical experts is crucial for growth factor supplementation. The source, whether bovine or not, is less important due to the fundamental biological actions of these proteins. The effects of the fundamental mechanisms, which are also seen in treatments using bovine colostrum-derived growth factors (listed in Table 2), can potentially be replicated by the patented products shown in Table 3.

Colostrum-derived growth factors are currently under research for their effectiveness and safety in treating various health conditions, focusing on their potential benefits in immune support, gastrointestinal health, and wound healing. Ongoing studies aim to clarify their role in improving gut integrity, enhancing immune response, and promoting tissue repair without significant adverse effects. In summary, the use of growth factors in the medical and nutritional supplementation fields represents a significant innovation that can support existing treatments and enable new therapeutic approaches. It is expected that this field will continue to expand, with growth factors being used in a wider range of applications in the future.

## 5. Conclusions and Future Outlook

BC is gaining increasing attention due to its various bioactive components. Among these components, growth factors stand out for their significant role in physiological processes. BC is a unique and powerful source of these development factors since it naturally contains them, unlike other supplements or synthetic substitutes.

It has been shown that these growth factors have many positive effects on the body. For instance, they can promote wound healing by encouraging tissue repair processes and cell proliferation. Growth factors may also be useful as therapeutic agents for diseases such as osteoarthritis or musculoskeletal injuries because they encourage the regeneration of deteriorating tissues. Unlike other sources, BC’s related growth factors are highly bioavailable and work together to improve these healing processes. Growth factors also play a critical role in strengthening the immune system by increasing the generation and activity of immune cells, which improves defense against infections and illnesses. The naturally occurring growth factors in BC promote a powerful immunological response, making it a better choice than manufactured immune boosters. Moreover, studies indicate that growth factors might be involved in more than just immune support. Some research suggests that they may serve as biomarkers for the diagnosis of diseases, demonstrating their involvement in pathological processes. Through comprehending the complex mechanisms by which growth factor’s function, researchers hope to open up new approaches to the diagnosis and treatment of disease.

The potential of BC as a helpful supplement for improving general health and well-being is highlighted by the complex impacts of growth factors. However, more research is needed to understand the effects of BC on health. Future studies can help us better understand the detailed mechanisms of action of growth factors and their effects on different health conditions. Furthermore, future research could investigate the best dosages and administration methods for enhancing the effects of colostrum-derived growth factors. Furthermore, we may uncover additional features of growth factors that could improve or broaden the benefits of colostrum ingestion. In this way, we can more comprehensively evaluate the role of BC in health and better understand its potential health benefits.

## Figures and Tables

**Table 2:** Clinical trials on growth factor supplementation.

Target Group	Study Design	Dose and Duration	Effect	Reference
Trained men	Pre-post-intervention study with repeated measures	60 g bovine colostrum Total: 4 weeks	BC does not affect blood IGF-1 or IGF binding protein-3 levels.	[66]
Human keratinocytes	Prospective experimental study	Not specified	BC supplementation induces mitogenic and motogenic effects on human keratinocytes, promoting cell proliferation and migration.	[67]
Guinea pigs	Prospective experimental study	Daily application of liquid formulation or gel formulation of the IM fraction on wounds Total: 23 days	Colostrum-derived whey immune fraction (IM fraction) inhibits collagen gel contraction in vitro, delays wound closure in full-thickness wounds, and minimizes residual scar formation in scar tissues.	[68]
Individuals with skin diseases	Cellular and molecular approaches on keratinocytes	Not specified	Promotes keratinocyte differentiation.	[69]
Human fibroblasts in vitro	Experimental study	BC at three concentrations (0.125%, 0.25%, 0.50%) for 8 weeks	Liposomal BC protects against telomere length erosion in fibroblasts under normal and oxidative stress conditions.	[70]
Keratinocyte cells in vitro	Experimental study	Fermented colostrum whey at concentrations of 100–400 μg/mL for observed effects	BC increased AQP-3 expression and cell proliferation via JNK and p38 MAPK activation.	[71]
Individuals with wounds	Pre-post-intervention study	Not specified	Enhanced wound healing properties.	[72]
Keratinocytes, melanocytes, and fibroblasts	Experimental study	Not specified	Effect of colostrum-derived exosomes: Prevented UV-induced damage, reduced melanin production, suppressed matrix metalloproteinase expression, increased cell proliferation, enhanced collagen production.	[73]
Patients with acne scars	Retrospective study	Fractional laser treatment: 120 days; recombinant bovine basic fibroblast growth factor(rbFGF): 300 IU/cm^2^ Total: 7 days	Improved skin barrier function, reduced lactic acid-induced stinging, enhanced stratum corneum integrity.	[74]
Fibroblasts, immortalized keratinocytes, Human vein umbilical endothelial cells	Experimental study	Rigemed D (BC containing 20 different growth factors and exosomes) doses: 1%, 1.5%, and 2% *v*/*v*; duration not specified	Rigemed D supplementation promotes cell proliferation, migration, and regeneration; exhibits antioxidant effects; and enhances angiogenesis	[75]
Critically ill, mechanically ventilated patients	Randomized controlled trial	30 g of bovine colostrum daily for 10 days	Increased serum levels of IGF-1; reduced incidence of diarrhea.	[76]
Rabbit flexor tendon cell populations	Experimental study	IGF-1: 10, 50, and 100 ng/mL PDGF-BB: 1, 10, and 50 ng/mL bFGF: 0.5, 1, and 5 ng/mL Duration not specified	Maximized tenocyte proliferation.	[77]
Injured rats	Prospective experimental study	0, 10, 100, or 1000 ng PDGF isoform B Total: 7 days	PDGF supplementation enhances tendon healing.	[78]
Epithelial and fibroblastic cells	Experimental study	2.5 mg/mL bovine milk for 48 h	Mitogenic extract from bovine milk promotes growth of mesodermal-derived cells.	[79]
Sheep fetuses with growth restriction	Randomized controlled trial in sheep	IGF-1: 360 μg Total: 128 days	Intrauterine administration of IGF-1 significantly increased fetal growth rate.	[80]
Meniscus	Experimental study	50 ng/mL bFGF and TGF-β3 Total: 8 weeks.	bFGF and TGF-β3 improved integration strength of meniscus repair constructs and electrospun PCL scaffolds. TGF-β3 increased proteoglycan content in the explants.	[81]
Tendon tissue	Experimental study	PDGF-BB and IGF-1 supplementation. Dose and duration not specified.	Enhanced cell attachment, alignment, viability, and metabolic activity.	[82]
Patients with skin burns	Prospective, randomized, double-blind clinical trial	EGF: 10 μg Total: 1.5 days	Accelerated rate of epidermal regeneration.	[83]
Patients with chronic wounds	Prospective, open-label, crossover trial	EGF: 10 μg Total: 6 months	Stimulation of wound healing.	[84]
Diabetic neuropathic or ischemic patients with high amputation risk	Non-controlled pilot study	EGF: 25 μg Total: 8 weeks	Reduction in diabetic lower limb amputation.	[85]
Hospitalized patients	Randomized, double-blind, within-patient, left/right, controlled trial	EGF: 10 μg in 0.1% silver sulfadiazine cream	Reduction of healing time of skin lesions in patients with pemphigus vulgaris.	[86]
Diabetic patients	Pilot study	Heberprot-P (EGF Based): 75 μg.	Exploration of clinical effects up to complete wound closure	[87]
Pediatric patients	Prospective clinical trial	EGF: 100 μg/kg Total: 6 weeks.	Improved carbohydrate absorption, increased tolerance to enteral feeds, reduced infection rates.	[88]
Mice with sarcopenia	Experimental study	12 µg pro-IGF-2/day Total: 7 days	pro-IGF-2 improves muscle regeneration by promoting satellite cell proliferation, angiogenesis, and inhibiting adipogenesis of PDGFRα+ cells	[65]

**Table 3:** Growth factor-based products.

Format	Growth Factor	Effect	Reference
Gel	EGF	Wound healing	[99]
Injection	EGF	Wound healing	[99]
Capsule	EGF	Wound healing	[99]
Injection	IGF-1	Growth disorders	[100]
Gel	PDGF	Bone repair and regenerative procedures	[101]
Topical Gel	EGF	Diabetic foot ulcer	[102]
Topical Gel	PDGF	Chronic wound healing (diabetic)	[103]
Collagen	TGF-β1	Anti-aging	[104]

## Data Availability

Not applicable.
